# Blinatumomab-driven T-cell activation in αβ and γδ T-cell subsets: insights from *in vitro* assays

**DOI:** 10.3389/fimmu.2026.1739493

**Published:** 2026-02-06

**Authors:** Miriam Kelm, Nourhan Nasr, Sonja Bendig, Dieter Kabelitz, Marta Lustig, Heiko Trautmann, Anna Laqua, Christian Peters, Daniela Wesch, Hans-Heinrich Oberg, Ottmar Janssen, Thomas Valerius, Claudia Dorothea Baldus, Alexander Scheffold, Monika Brüggemann, Guranda Chitadze

**Affiliations:** 1 Medical Department II, Hematology and Oncology, Christian-Albrechts University of Kiel and University Hospital Schleswig-Holstein, Kiel, Germany; 2 Institute of Immunology, Christian-Albrechts University of Kiel and University Hospital Schleswig-Holstein, Kiel, Germany; 3 Clinical Research Unit Towards a Cure for Adults and Children with Acute Lymphoblastic Leukemia (CATCH ALL) (KFO 5010), Funded by the Deutsche Forschungsgemeinschaft (DFG, German Research Foundation), Kiel, Germany; 4 University Cancer Center Schleswig-Holstein (UCCSH), University Hospital Schleswig-Holstein, Kiel, Germany; 5 Medical Department II, Division of Stem Cell Transplantation and Cellular Immunotherapies, University Hospital Schleswig-Holstein and Christian-Albrechts-University of Kiel, Kiel, Germany; 6 Technische Universität Berlin, Institute for Biotechnology, Berlin, Germany

**Keywords:** adoptive T-cell therapy, BCP ALL, bispecific antibody, blinatumomab, immunotherapy, γδ T cells, Zoledronate

## Abstract

**Introduction:**

Blinatumomab (BLN) is a bispecific T-cell engager that has revolutionized the treatment of B-cell precursor acute lymphoblastic leukemia (BCP-ALL), significantly improving outcomes in both adults and children. By simultaneously binding to CD19 on B cells and CD3 on T cells, BLN triggers target cell-dependent T-cell activation, resulting in the cytolysis of CD19^+^ BCP-ALL cells. Despite the remarkable clinical advancements achieved with BLN, the immunological mechanisms underlying treatment response or failure remain poorly characterized. γδ T cells are attractive candidates for adoptive T-cell therapy due to potent cytotoxicity, capacity to present antigens, broad lysis of different tumor entities, and low alloreactivity. Because γδ T cells can also be redirected by BLN, we systematically studied BLN-driven effector functions *in vitro* in conventional αβ and unconventional γδ T cells from healthy donors.

**Materials and methods:**

We evaluated cytotoxicity and cytokine/effector release in freshly isolated and *in vitro*-expanded αβ and γδ T cells from healthy adults against CD19^+^ BCP-ALL cell lines (NALM-6, HAL-01), and profiled dynamic phenotypic alterations by multiparametric flow cytometry.

**Results:**

CD19^+^ targets were consistently reduced in the presence of BLN. Freshly isolated αβ, especially CD8^+^, displayed superior BLN-mediated effector functions as compared to γδ T cells, with donor-dependent variability in γδ killing. Notably, zoledronate-expanded Vγ9Vδ2 γδ T-cell lines achieved cytotoxicity comparable to PHA-expanded αβ cells. However, γδ T-cell-killing benefited from higher BLN concentration when challenged with high tumor load. In these *in vitro* healthy-donor T-cell cultures, BLN induced CD3 down-modulation in αβ T cells but not in γδ T cells, and αβ cultures released higher soluble Fas ligand, findings consistent with stronger early activation and suggestive of increased susceptibility to activation-associated apoptosis/AICD. Exploratory targeted single-cell transcriptomics (one donor) supported a pronounced activation/exhaustion program in αβ T cells and a comparatively stable effector-memory profile with low checkpoint expression in γδ T cells.

**Discussion:**

Together, these *in vitro* data reveal subset-specific BLN responses and support the hypothesis that ex vivo-expanded Vγ9Vδ2 γδ T cells could complement BLN-mediated cytotoxicity, particularly under conditions of higher CD19 density and lower target burden. These findings provide a mechanistic framework for future testing of γδ T-cell/BLN combination strategies in patient-derived models and clinical studies.

## Introduction

The outcome of B-cell precursor acute lymphoblastic leukemia (BCP-ALL) has improved significantly over the last decade due to personalized therapy protocols and novel therapeutic strategies, including mono- or bispecific antibodies, antibody-drug conjugates, and genetically modified CAR T cells (1, 2). Nevertheless, relapse remains common, and more than 20% of patients still succumb to the disease. Outcomes are notably poorer in adults than in children, with five-year overall survival rates of around 90% in pediatric cohorts but only 40-60% in adults (3–6). This highlights the critical need to understand the mechanisms underlying therapy failure and to further optimize clinical outcomes.

Blinatumomab (BLN), a bispecific T-cell engager linking CD3 on T cells with CD19 on B cells, facilitates the formation of immunological synapses, leading to T-cell activation, expansion, and targeted lysis of CD19^+^ cells (2, 7). Since its initial FDA approval in 2017 for relapsed or refractory B-cell precursor ALL (R/R BCP-ALL) in both adults and children, BLN has demonstrated remarkable clinical efficacy (8–11). Nonetheless, many patients relapse or fail to achieve long-term remission after BLN therapy in R/R or frontline in minimal residual disease (MRD) settings (9, 12, 13). Approximately 25% of these failures are due to loss of target antigen; the rest of the relapse cases however are due to impaired T-cell functionality (14). In heavily pretreated patients, such dysfunction may arise from prior chemotherapy regimens (15), from high leukemic burden (8, 13) or from persistent T-cell stimulation due to BLN’s administration as a 28-day continuous infusion, required due to its short half-life (16, 17).

Given these limitations, exploring novel strategies to enhance BLN-mediated cytotoxicity through complementary therapeutic approaches is essential. One promising approach involves the transfer of γδ T cells from unrelated donors, a minor yet highly potent subset of T cells characterized by CD3-associated TCR γδ expression and intrinsic anti-tumor cytotoxicity irrespective of HLA-matching (18–20). Adoptive γδ T-cell therapy is attractive because γδ T cells recognize tumor cells through stress-induced ligands without relying on HLA presentation. γδ T cells have a reduced risk of graft-versus-host disease (GvHD) and are suitable for allogeneic application across diverse patient populations (21–23).

Peripheral γδ T cells constitute up to 5-10% of circulating T cells, predominantly represented by the Vγ9Vδ2 γδ T-cell subset (18, 24, 25). These cells respond robustly to pyrophosphates (“phosphoantigens”) frequently accumulating in tumor cells, and their selective activation and expansion can be reliably induced using zoledronic acid (Zole) or synthetic phosphoantigens, thus facilitating clinical-grade ex vivo expansion (26, 27). γδ T-cell activation depends on interactions with BTN3A1 and BTN2A1 (28, 29). Additionally, γδ T cells can function as antigen-presenting cells, cross-presenting antigens to αβ T cells and further enhancing immune responses (30, 31). Their broad tumor-recognition capacity, combined with independence of HLA-restriction, positions γδ T cells as highly favorable candidates for adoptive cell therapies across diverse malignancies, as currently explored in numerous clinical studies utilizing expanded Vδ1 and Vδ2 T-cell subsets (19). Clinical safety of allogeneic Vγ9Vδ2 γδ T-cell immunotherapy has already been documented, with prolonged survival reported in patients with late-stage lung or liver cancer (32).

Our own results suggest prognostic value of γδ T cells in ALL, with increased levels correlating to improved MRD responses post-chemotherapy (33). Limited preclinical evidence, supports the therapeutic potential of combining adoptively transferred γδ T cells with CD3-engaging antibodies, including BLN (34). However, their ability to augment BLN-mediated cytotoxicity in adoptive immunotherapy settings has not been fully evaluated. Moreover, γδ T cells may follow distinct exhaustion pathways compared to αβ T cells (35, 36), but this has not been investigated in the context of BLN.

In this *in vitro* study, we systematically investigated BLN-mediated changes in the effector functions of *ex vivo* expanded healthy donor-derived γδ T cells compared to αβ T cells. We assessed their phenotypic and functional fitness under conditions mimicking variable tumor burdens typically encountered at diagnosis or during therapy. Our approach combined functional assays, multiparametric phenotyping, and single-cell transcriptomics to dissect how γδ T cells kill CD19^+^ targets in the presence of BLN. A deeper understanding of these mechanisms may inform novel therapeutic strategies to improve outcomes of BLN-treated patients with BCP-ALL.

## Materials and methods

### Cell cultures

Peripheral blood mononuclear cells (PBMCs) were isolated from leukocyte concentrates of healthy adult blood donors obtained from the Institute of Transfusion Medicine (Institute for Transfusion Medicine, University Hospital Schleswig-Holstein Campus Kiel) using Ficoll density gradient centrifugation (Merck, Darmstadt, Germany). Ethical approval was granted by the institutional ethics review board of the University Medical Faculty Kiel (approval number D405/10, D479/18, D546/16). The research was conducted in accordance with the Declaration of Helsinki. CD4^+^, CD8^+^, and γδ T cells or when required entire CD3^+^ T cells were purified from PBMCs by magnetic-activated cell sorting (MACS) using respective negative selection kits according to the manufacturer’s instructions (Miltenyi Biotec, Bergisch Gladbach, Germany).

To establish αβ T-cell lines, PBMCs were stimulated with Phytohemagglutinin A (PHA; 0.5 µg/ml; Thermo Fisher Scientific, Waltham, MA, USA) and recombinant interleukin-2 (rIL-2; 100 U/ml; Novartis, Basel, Switzerland) in culture medium which consists RPMI 1640 medium (Thermo Fisher Scientific) supplemented with penicillin (100 U/ml), streptomycin (100 µg/ml), and 10% heat-inactivated fetal bovine serum (FBS; Thermo Fisher Scientific), hereafter referred to as culture medium, following previously established protocols (37, 38). Vδ2 T cells were expanded using Zoledronate (Zole, 2.5 µM; Novartis) and rIL-2 (50 IU/ml) to establish short-term activated Vδ2 T-cell lines as previously reported (39). IL-2 was added every 2-3 days and cultures were split after day 6 or 7. After 14 days, non-viable cells were removed via Ficoll density centrifugation when necessary. The purity of expanded cell lines was assessed at day 14 by flow cytometry.

The human B-cell precursor ALL cell lines NALM-6 and HAL-01 obtained from DSMZ and maintained in required culture medium according to manufacturer’s instructions and utilized as target cells in co-culture assays.

### Co-culture experiments

Freshly isolated CD4^+^, CD8^+^, and γδ T cells were co-cultured with HAL-01 cells at an effector−to−target (E:T) ratio of 5:1, with or without BLN (20 ng/ml) for up to 7 days (40). T-cell numbers were assessed via manual counting at day 3 and day 7 and analyzed for their composition and phenotypes by flow cytometry at day 3. Supernatants were collected at specified day 3 and day 7, stored at −20°C, and analyzed via ELISA as described below. *In vitro* expanded PHA-activated αβ and Zole-activated γδ T cells were co-cultured with NALM-6 or HAL-01 target cells at various E:T ratios in the presence or absence of BLN (20 ng/ml or 0.5 ng/ml) for 24 hours or 3 days. PBMCs from 6 healthy donors were co-cultured for 7 days with the CD19^+^ BCP-ALL cell line HAL-01 at different effector-to-target (E:T) ratios: 1:1 (high CD19 load), 5:1 (medium CD19 load), or without addition of exogenous target cells (low CD19 load, autologous B cells only), in the presence of in the absence of BLN (20 ng/ml). Cells were harvested and analyzed on days 0, 3, and 7 by multiparametric flow cytometry. Effector cells at fixed numbers were cultured with varying amounts of target cells through all co-culture experiments. Recombinant human IL-2 (50 IU/ml) was added to all co-cultures at baseline and replenished every 48 h (no other exogenous cytokines were used). Media volume lost to sampling was replaced with fresh complete medium containing the same IL-2 concentration.

### Multiparametric flow cytometry

Purity of PHA- and Zole- expanded αβ and γδ T-cell cultures and MACS-isolated T-cell populations were assessed by flow cytometry using anti-CD3, CD4, CD8, γδ TCR, and αβ TCR monoclonal antibodies. Only populations with >80-90% purity were used in co-culture assays. For all analyses, cells were gated on singlets and live cells defined based on scatter properties, followed by CD3^+^ lymphocytes and subsequent αβ/γδ and memory−subset gates (**Supplementary Table 1**, gating strategy depicted in **Supplementary Figures 1**, **2**).

B-cell depletion in BLN-activated PBMCs or in co-cultures was analyzed on BD FACS Canto using antibodies directed against CD19 and CD3 or at Cytek Northern Lights cytometer using a 22-color antibody panel also incorporating anti-CD3 and anti-CD19 with the following antibodies directed against CD4, CD8, CD25, CD27, CD28, CD38, CD45, CD45RA, CD56, CD95, Annexin V, CCR7, DNAM-1, HLA-DR, PD-1, γδ TCR, TIGIT, TIM-3, Vδ2 γδ TCR and 7-AAD.

Selected samples undergoing single-cell RNA sequencing (BLN-treated and untreated samples of single healthy donor T cells) were analyzed using antibodies against CD3, CD4, CD8, CD16, CD19, CD22, CD24, CD45, CD45RA, CD56, CCR7, DNAM-1, γδ TCR and Vδ2 γδ TCR. Samples were analyzed on a BD FACSLyric. Detailed information of used antibody panels and used clones or formats is provided in **Supplementary Table 2**.

### Enzyme-linked immunosorbent assay

Cell culture supernatants were analyzed for Interferon-γ (IFNγ; DIF50C), Granzyme B (GrzB; DGZB00), Perforin (QK8011), Granulysin (Gnly; DY3138), Perforin (Prf; QK8011) and soluble Fas-Ligand (sFAS-L; DFL00B) using sandwich ELISA kits from R&D Systems, Minneapolis, USA according to the manufacturer’s instructions. Absorbance was measured at 450 nm using a microplate reader (Tecan Group Ltd, Männedorf, Switzerland) in the Institute of Immunology Kiel.

### Determination of CD19 antigen-density

To quantify CD19 antigen density on the cell surface of BCP ALL cell lines (NALM6 and HAL-01), the specific antibody binding capacity (SABC) was determined using the purified anti-human CD19 antibody (clone HIB19) and the QIFIKIT^®^ (DAKO, Glostrup, DK) by flow cytometry according to the manufacturer’s instructions in the Division of Stem Cell Transplantation and Cellular Immunotherapies.

### BD Rhapsody single-cell RNA-seq

BD Rhapsody Single-Cell Analysis System (BD, Biosciences) were utilized using a targeted approach with human T-cell Expression Panel commercially available from BD Biosciences and covering 259 genes, commonly expressed in human T cells (Cat ID 633751, **Supplementary Table 3**). For this, PBMCs from one healthy donor were cultured for 72 hours with rIL-2 (50 IU/ml) + BLN (20 ng/ml) or with rIL-2 alone as a control. Afterwards, CD3^+^ cells were negatively isolated (human MACS Pan T-cell isolation Kit, Miltenyi Biotec), labeled using BD Single-Cell Multiplexing Kit and AbSeq (antibody-labeled with oligos) reagents targeting CD4, CD8, CD25, CD45RA, CD127, CCR7, and TCRγδ (**Supplementary Table 2**). Cells from BLN-treated and control samples were labeled with Sample Tags following the manufacturer’s instructions (Single Cell Labelling with the BD™ Single-Cell Multiplexing Kit and BD™ AbSeq Ab-Oligos (ID: 214419 Rev. 2.0)). 10, 000 single cells were load on a cartridge with preloaded beads to capture RNA of single cell, followed by cDNA synthesis using the BD Rhapsody Express Instrument and Scanner following BD protocols (Single Cell Capture and cDNA Synthesis with the BD Rhapsody™ Single-Cell Analysis System (210966 Rev. 1.0)). cDNA libraries of target transcripts, Samples Tags and Abseqs were prepared using the Manufactures instructions, following mRNA Targeted, Sample Tag, and BD™ AbSeq Library Preparation with the BD Rhapsody™ Targeted mRNA and AbSeq Amplification Kit (214508 Rev. 3). The final libraries were quantified using a Qubit Fluorometer with the Qubit dsDNA HS Kit (ThermoFisher) and the size-distribution was measured using the Agilent high sensitivity D5000 assay on a TapeStation 4200 system (Agilent Technologies). Sequencing was performed in paired-end mode (2x75 cycles) on NextSeq 500 System (Illumina) with NextSeq 500/550 Mid Output Kit, generating approximately 1.3 billion reads. Data processing utilized the Seven bridges pipeline BD Rhapsody™ Targeted Analysis Pipeline - Revision: 0. Generated demultiplexed matrices of ScRNA-seq UMI count were imported to R 4.3.3 and gene expression data analysis was performed using the R/Seurat package 5.3.0 (41). To remove doublets and cell fragments, we further fitted a linear model of detected genes versus transcript count per sample and excluded deviating outliers (0.5 - 5% of cells, depending on sample quality). Additionally, we removed genes that were expressed in less than ten cells. Before downstream analysis, LogNormalization (Seurat function) was applied separately to the RNA and AbSeq assays. The data were then scaled regressing for total UMI counts and principal component analysis (PCA) was performed. RNA and AbSeq data were integrated using weighted nearest neighbors (WNN) based on the first 8 RNA and first 6 AbSeq principal components. UMAP and clustering were performed on the resulting WNN graph. For two-dimensional data visualization we performed UMAP based on the first 20 dimensions of the resulting WNN graph. The cells were clustered using the Louvain algorithm based on the first 20 dimensions with a resolution of 0.8. Cluster annotation was based on gene expression signatures (top 5 characteristic genes per cluster) and AbSeq expression (**Supplementary Table 4**).

### Statistical analysis

For paired comparisons of matched donors, we used two−tailed paired t−tests. For comparisons between non−overlapping donor sets, we used two-tailed unpaired t-tests. For multiparameter immunophenotyping, p-values were adjusted for multiple testing using the Benjamini-Hochberg false discovery rate (FDR) method across the set of markers/subsets tested within each comparison. In all figures, n denotes the number of independent donors (biological replicates). No multiple-comparison adjustment was applied to ELISA readouts because each analyte was analyzed as an independent, pre-specified endpoint (single assay per analyte), whereas FDR correction was applied to high-dimensional flow-cytometry marker panels. Statistical significance was set at p<0.05 (*), p<0.01 (**), p<0.001 (***). Analyses were performed using GraphPad Prism 8.4.3 and RStudio 4.3.3.

## Results

### BLN potentiates cytotoxicity of fresh αβ and γδ T cells, but αβ T cells dominate early cytotoxicity

**Figure 1:**
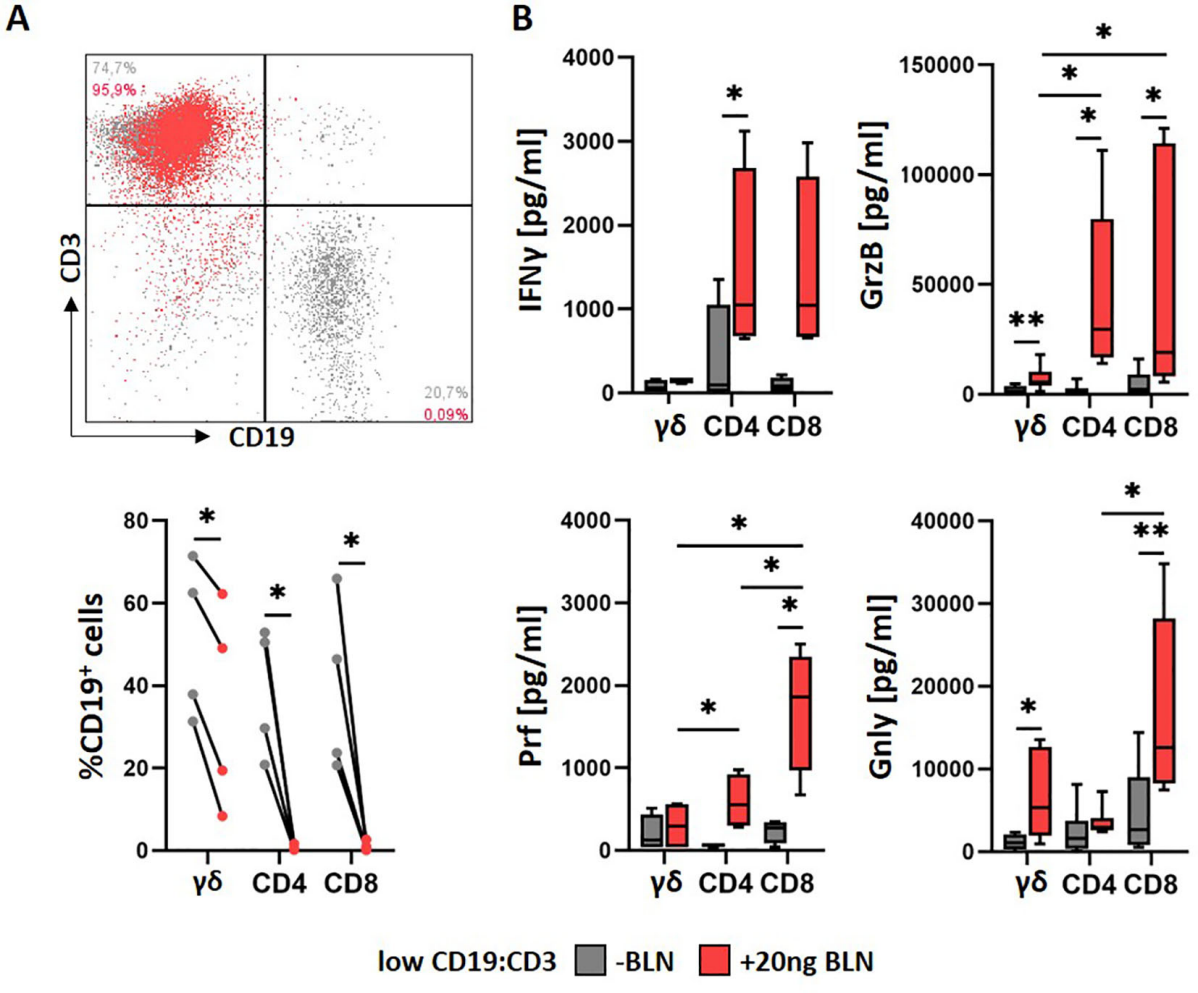
BLN-mediated T-cell responses against CD19^+^ malignant B-cell line. **(A)** BCP-ALL cell lines in the presence (red) or absence (grey) of BLN for three days were co-cultured at an effector-to-target ratio of 5:1 (low CD19:CD3) with freshly isolated T cells (γδ, CD4^+^ and CD8^+^ αβ T cells) from healthy donors (n = 4). T- and B-cell frequencies in the coculture were assessed by staining for CD3 and CD19, as exemplary shown for CD8^+^ αβ T cells (upper panel). **(B)** The release of IFNγ (n = 4), GrzB (n=7) and Prf (n = 4) into supernatants of cocultures in the presence (red) or absence (grey) of BLN was assessed via ELISA after three days, and the release of Gnly (n = 6) was analyzed after seven days. Paired two−tailed t−tests were used for donor−matched ± BLN comparisons. *p<0.05 and **p<0.01.

We first evaluated BLN-induced killing of CD19^+^ target cells by freshly isolated γδ, CD4^+^ and CD8^+^ αβ T-cell populations. Isolated T-cell populations (γδ, CD4^+^ and CD8^+^ αβ) were co-cultured with the CD19^+^ malignant BCP ALL cell line HAL-01 at an effector-to-target ratio of 5:1 in the presence of BLN at concentration of 20 ng/ml, previously demonstrated to effectively induce immunological synapse formation (40). At day three, target CD19^+^ cells were consistently reduced in all co-cultures in the presence of BLN, as measured by assessing residual CD19^+^ cells using flow cytometry (**Figure 1A**, top panel shown for CD8^+^ αβ T cells). However, γδ T cells displayed lower cytolytic activity compared to CD8^+^ and CD4^+^ αβ T cells, demonstrated by persistence of CD19^+^ target cells in γδ and not in αβ T-cell cultures in the presence of BLN (**Figure 1A**, bottom panel). To account for donor−dependent background killing, we additionally quantified BLN−specific target elimination by normalizing each donor to its no BLN control (**Supplementary Figure 3A**): residual CD19^+^ cells in the no BLN condition were set to 100%, and the corresponding +BLN condition was expressed relative to this baseline (i.e., a donor-normalized fold-reduction metric). This normalization did not change the overall conclusion that CD8^+^ and CD4^+^ αβ T cells mediated stronger BLN-dependent killing than γδ T cells.

ELISA assays revealed significantly increased secretion of Interferon-γ (IFNγ) and cytotoxic effector molecule Granzyme B (GrzB), Perforin (Prf) and late mediator of cytotoxicity Granulysin (Gnly) (42). At day 3 in BLN-treated cultures, with αβ T cells releasing consistently higher levels than γδ T cells. Prf levels were highest in CD8^+^ T-cell cultures at day 3, whereas Gnly levels, assessed after seven days, were significantly elevated in both CD8^+^ and γδ T-cell cultures treated with BLN (**Figure 1B**).

Overall, BLN enhanced cytotoxic potential of freshly isolated γδ and αβ T-cell populations. However αβ T-cell populations demonstrated superior BLN-mediated cytolytic activity compared to γδ T cells, with CD8^+^ T cells having the potentially highest cytotoxic potential, as indirectly indicated by perforin secretion (43).

### Activated γδ T cells match αβ cytotoxicity under favorable antigen conditions

**Figure 2:**
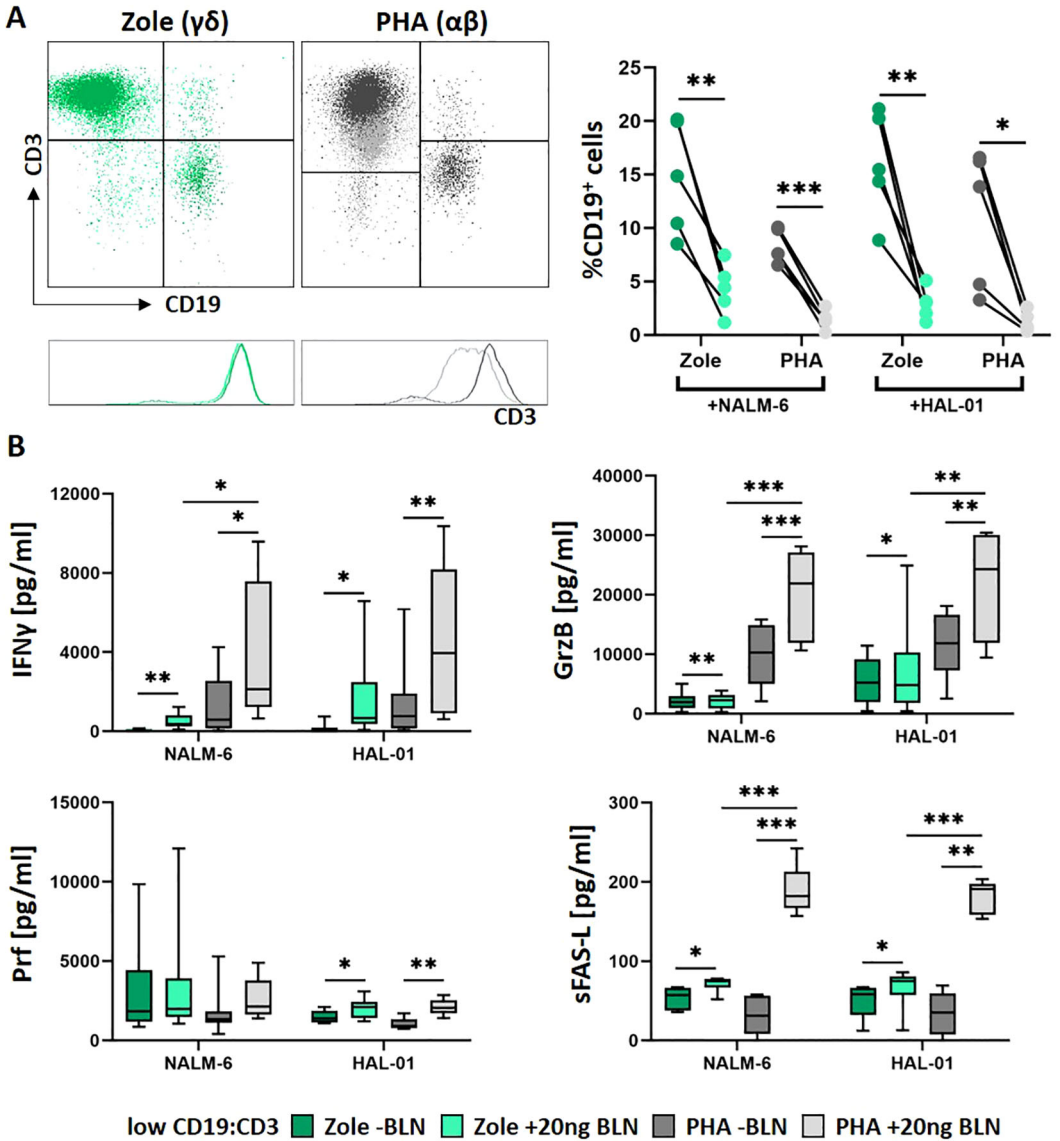
BLN-induced release of cytotoxic effector molecules from Zole- expanded γδ and PHA-expanded αβ T cells. *In vitro* PHA-expanded aβ (grey) and Zole-expanded γδ T cells (green) were cocultured with different malignant B-cell lines (NALM-6, HAL-01) in the presence or absence of BLN for 24 h with an effector-to-target ratio of 5:1 (low CD19:CD3) (n = 5). **(A)** Exemplary staining at 24h, defining residual CD19+ cells and CD3+ in the co-culture in presence of BLN and PHA or Zole activated T cells are shown. **(B)** The release of IFNγ (n = 6-10), GrzB (n = 6-10), Prf (n = 5-10) and sFAS-L (n = 5-6) was analyzed in respective coculture supernatants of BLN-treated or untreated cells via ELISA. Paired two−tailed t−tests were used for ± BLN comparisons within matched donors. For PHA vs Zole comparisons, paired tests were used when donor−matched; otherwise unpaired tests were applied. *p<0.05, **p<0.01, ***p<0.001.

Given the known cytotoxic potential of Zole-activated Vδ2 T cells and their potential clinical application (19, 44), we next assessed BLN-dependent killing using Zole−expanded Vγ9Vδ2 γδ T-cell lines. Using the same flow-based readout (residual CD19^+^ cells), we compared their activity with PHA-expanded αβ T cells against HAL−01 and NALM−6, which differ in CD19 surface density (HAL−01 ca. 2.5−fold higher CD19 antigen density than NALM−6; **Supplementary Figure 4**). We studied release of cytokine mediators/effector molecules at 24 hours anticipating stronger effector functions (**Figure 2B**) in the presence of BLN by Zole-expanded γδ T cells (**Figure 2A**, green) and compared it with effector functions mediated by PHA-expanded αβ T cells (**Figure 2A**, grey) against two BCP-ALL cell lines, HAL-01 and NALM-6, with high and low expression CD19-patterns respectively (**Supplementary Figure 4**). At an effector-to-target ratio of 5:1 and BLN concentration of 20 ng/ml, both γδ and αβ activated cultures resulted in significant CD19^+^ reduction. However, PHA-expanded αβ T cells induced a slightly more pronounced B-cell killing with complete clearance of CD19^+^ cells. Target cells still persisted in Zole-expanded γδ T cells after 24 hours at low levels, particularly in a case of NALM6 (residual CD19^+^ cells at median value of 4.4% of co-culture), expressing lower levels of CD19 compared to HAL-01 (**Supplementary Figure 4**). Interestingly, we observed CD3 downregulation, that selectively occurred in PHA-expanded αβ T cells in the presence of BLN, whereas Zole-expanded γδ T cells maintained stable CD3 expression (**Figure 2A**, bottom panel with exemplary histograms). BLN-independent T-cell responses were also observed, as indicated by partial B-cell elimination in the absence of BLN occurring in a donor-dependent manner, with PHA-expanded αβ T cells (dark grey) outperforming Zole-expanded γδ T cells (dark green). BLN-dependent T-cell responses were also calculated by normalized residual CD19-cells to their counts in the absence of BLN and resulted in the same outcome (**Supplementary Figure 3B**).

Both expanded γδ and αβ T-cell lines exhibited increased secretion of IFNγ, GrzB, soluble FAS ligand (sFAS-L), and Prf following BLN stimulation in the presence of tumor cells. IFNγ and GrzB were lower in Zole-expanded cultures compared to PHA-expanded cultures (**Figure 2B**). Interestingly, high levels of sFAS-L, known to occur during activation induced cell death (AICD) expected in case of excess of T-cell activation (45, 46), was higher in the presence of PHA-expanded αβ T cells compared to Zole-expanded γδ T cells. Thus, when comparing Zole and PHA-activated T-cell cultures, αβ T-cells exhibit superior cytotoxicity; however, release higher levels of soluble FAS-L and potentially undergo CD3 downregulation in contrast to γδ T cells.

### Target antigen density/tumor burden jointly impact γδ efficacy; higher BLN dose preferentially benefits γδ under stress

**Figure 3:**
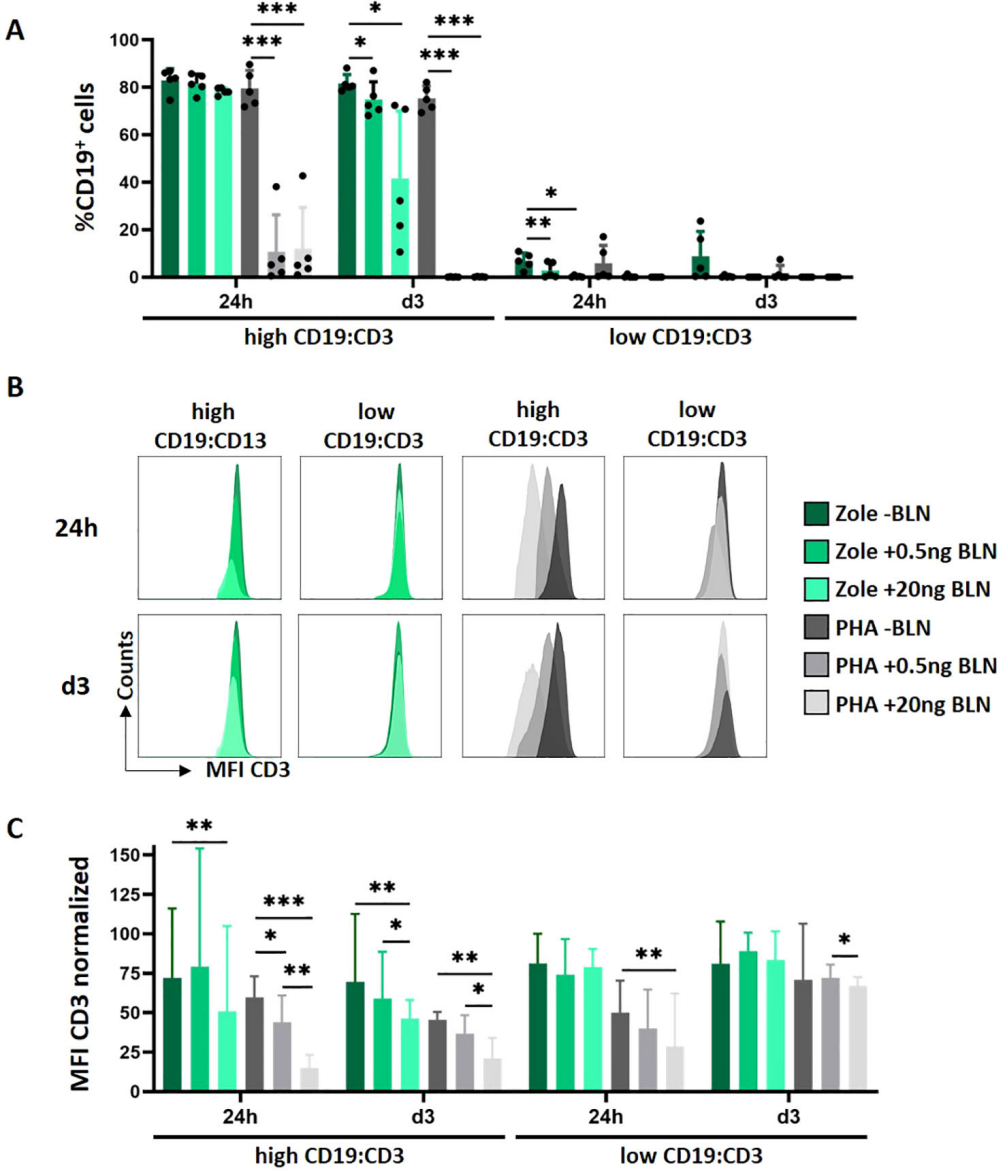
Impact of tumor burden and BLN concentration on T-cell-mediated cytotoxicity in Zole (γδ) - and PHA (αβ)-expanded T cells. **(A)** Median B-cell decline of HAL-01 cells from Zole- (green) and PHA-expanded T cells (grey) healthy donors (n = 5). Individual values are represented by dots. **(B)** Representative example of CD3-expression in γδ and αβ T-cell cultures, following BLN-mediated activation. **(C)** Normalized CD3 MFI of different culture conditions (n = 5). High = High Tumor load (E:T 1:5), Low = Low Tumor load (ET 5:1). CD3 MFI was normalized to the corresponding unstained control (CD3 MFI/unstained MFI). Paired two−tailed t−tests were used for donor−matched ± BLN comparisons. *p<0.05, **p<0.01, ***p<0.001.

In clinical setting, response to BLN is largely dependent on ALL blast counts (8). Blinatumomab has higher affinity to CD19 and lower affinity to CD3 (47). BLN binds to CD19 and acts as activation matrix for T cells, that can detach and kill up to 5‐10 target cells within 9 hours (Serial killing) (48, 49). In the absence of CD19^+^, no T-cell activation takes places (47). Thus, CD19 target expression is relevant for BLN mediated T-cell activation and target antigen density/target cell load, as well as the concentration of BLN might impact T-cell signaling and activation (8). Thus, we next explored how varying the effector-to-target ratio and BLN concentration influenced γδ T-cell cytotoxicity. Using the HAL-01 cell line, which exhibits higher CD19 antigen density than NALM-6 (ca. 2.5−fold; **Supplementary Figure 4**), we cultured Zole-expanded γδ T-cell lines under low target burden (E:T 5:1; MRD-like) and high target burden (E:T 1:5; diagnosis/relapse−like) conditions (**Figure 3**). BLN was tested at 20 ng/ml and at concentration of 0.5 ng/ml, close to steady-state serum level reported in patients (50). Residual target (CD19^+^) cells and T-cell composition/phenotypes linked with T-cell functionality were evaluated after 24 hours and following three days by 22-color spectral flow cytometry (**Supplementary Table 2**).

Under low target burden (E:T 5:1; low CD19:CD3), both γδ (Zole-expanded) and αβ (PHA-expanded) cultures efficiently eliminated CD19^+^ target cells (**Figure 3A**, right panel). Under high target burden (E:T 1:5; high CD19:CD3, **Figure 3A**, left panel), αβ T-cell cytotoxicity appeared near-maximal already at 24 h and did not increase further with higher BLN, whereas γδ T-cell killing improved with 20 ng/ml BLN, particularly by day 3 (3/5 donors; **Figure 3A**, left panel, green bars). BLN-specific target cell elimination based on donor-normalized residual target cells yielded the same pattern (**Supplementary Figure 3C**).

Consistent with previous findings, marked CD3 downregulation was observed predominantly in αβ T cells upon BLN treatment, especially at high tumor burden conditions (**Figures 3B, C**; **Supplementary Figure 5**). Because CD3 down-modulation occurred only at high target burden, and spared γδ T cells while primarily affecting αβ T cells under identical BLN and staining conditions, this pattern is consistent with CD3 internalization after strong T-cell stimulation rather than epitope masking (51), supported by the condition-matched unstained controls and representative histograms (**Supplementary Figure 5**).

### Zole-expanded γδ T cells retain a stable effector memory phenotype with low checkpoint expression under BLN

The differential CD3 modulation observed in γδ and αβ T cells suggested distinct BLN-mediated signaling through CD3, which we hypothesized would result in phenotypic and functional differences, affecting their fitness and potentially susceptibility to AICD, as supported by the high levels of sFAS-L detected in αβ T-cell cultures. Of note, sFas-L reflects ADAM10/17−mediated shedding of the pro-apoptotic membrane FasL and elevated sFasL in CD8^+^ cultures are here interpreted as a biomarker of strong re-stimulation and shedding, not as a direct driver of AICD (45, 46, 52).

**Figure 4:**
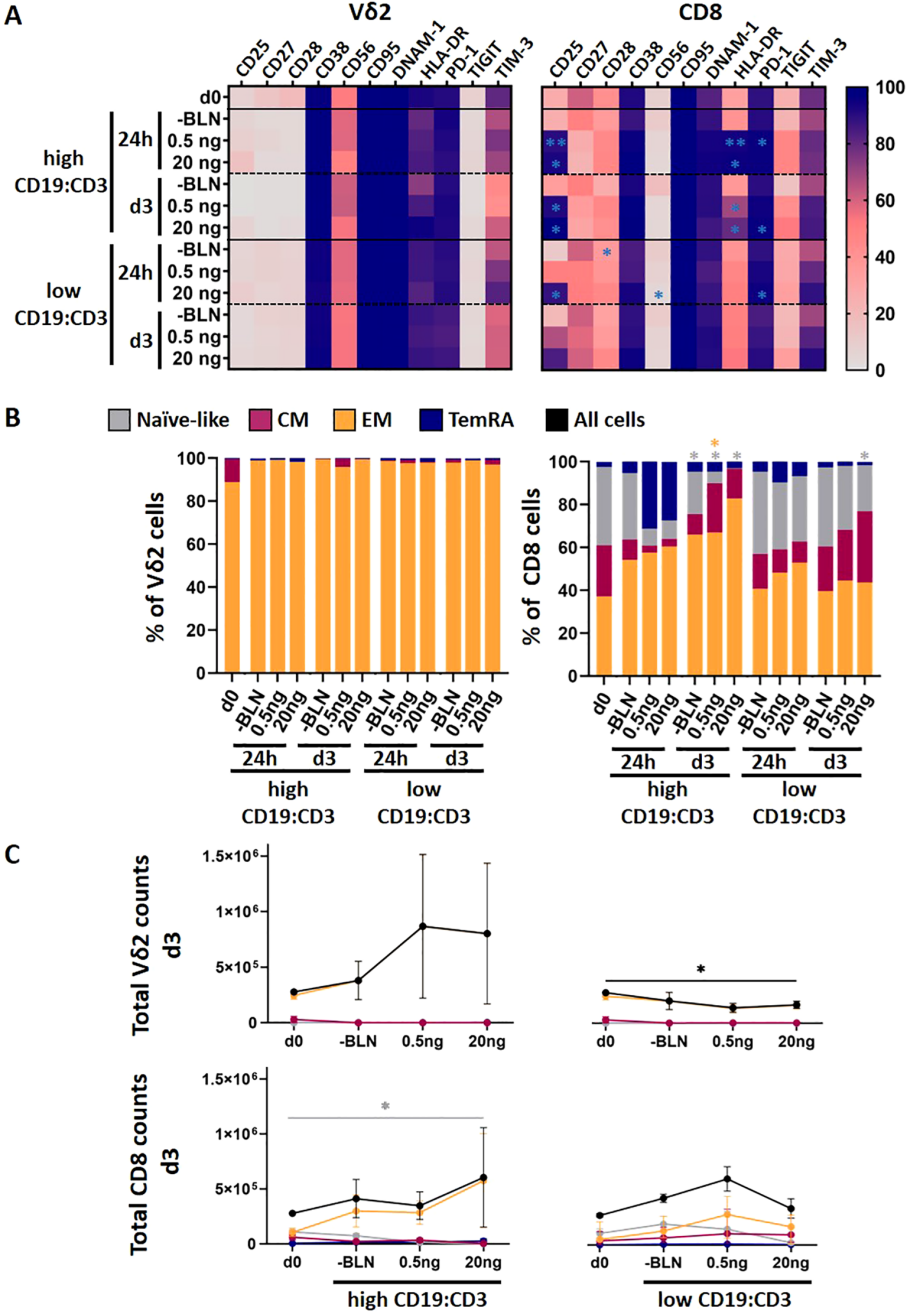
Phenotypic differences between PHA and Zole activated αβ and γδ T cells. **(A)** Mean Marker Expression on Zole-expanded Vδ2 and PHA-expanded CD8 T cells at baseline, at day 1 and day 3 following co-culture with high (E:T 1:5) and low tumor load (n = 5). **(B)** Mean values of EM, CM, Naïve and TEMRA cells of CD8 and Vδ2 cells. **(C)** Absolute cell counts of EM, CM, Naïve and TEMRA cells of CD8 and Vδ2 cells at day 0 and day 3. Data includes 5 matched healthy donors, depicted as mean value with SEM. Paired two‑tailed t‑tests were used for donor‑matched comparisons between baseline and co-culture conditions and p−values were adjusted using Benjamini-Hochberg FDR across markers within each comparison. *p<0.05, **p<0.01, ***p<0.001.

To investigate this, we compared the composition and phenotype of Zole- and PHA-expanded T cells from 5 healthy donors at baseline (prior to co-culture) and analyzed their numbers and phenotypic changes at day 1 and day 3 using spectral flow cytometry (**Figure 4**, **Supplementary Figure 6**). At baseline prior co-culture (d0), after 14 days of expansion, T-cell composition differed markedly: Zole-expanded cultures consisted expectedly of Vδ2 γδ T cells (median 90%, range 80-97%), while PHA cultures comprised both CD4^+^ and CD8^+^ αβ T cells with median proportions of 14% (range 6%-31%) and 66% (range 63%-84%), respectively (**Supplementary Table 1**).

Zole-expanded Vδ2 γδ T cells displayed a highly homogeneous effector memory (EM) phenotype (CD27^-^CD45RA^-^), consistent with previous observations (39, 44). Their absolute cell counts did not significantly change during three days of co-culture across different BLN concentrations at high ALL load (**Figure 4C**, left top panel, black line). We observed slight decrease in absolute counts in low CD19:CD3 conditions (**Figure 4C**, right top panel, black line). Day 14 Zole-expanded γδ T cells expressed high levels of activation markers (CD38, HLA-DR, DNAM-1, CD56) while showing lower or absent levels of exhaustion markers such as TIGIT (baseline, d0). During co-culture there was not significant change of their phenotype compared to baseline (**Figure 4A**). TIGIT negativity on day 14 cultures goes in line with a reported transient peak around day 4 and declining by day 7 in Zole-expanded T-cell cultures (35).

In contrast, PHA-expanded CD8^+^ αβ T cells were more heterogeneous with naïve and memory subsets at baseline. Upon BLN stimulation, especially in the context of high tumor load, naïve cells declined and EM and TEMRA (CD27^-^CD45RA^+^) subsets increased after 24 hours, with EM cells becoming dominant by day 3 (**Figure 4B**). This was reflected by an increase in total EM counts in the presence of high tumor load at day 3 (unadjusted p=0.019; FDR−adjusted p=0.09, trend), as single dominant population. In cultures with lower CD19^+^ burden (**Figure 4C**, left bottom panel), in line with less activation provided by low target cell density, naïve CD8^+^ T cells transitioned toward both EM and central memory (CM) phenotypes. Interestingly absolute counts in the presence of low target cells and lower BLN amount was higher compared to high target density and low BLN concentration. Phenotypic shifts observed in PHA-expanded CD4^+^ αβ T cells following BLN are shown as **Supplementary Figure 6**.

Phenotypically, PHA-expanded CD8^+^ αβ T cells showed strong activation in response to BLN, characterized by increased expression of CD25 and HLA-DR in line to observed differentiation from naïve to memory phenotypes. TIGIT expression was markedly high in αβ T cells at baseline. We also attempted to study differences in AICD at 24h or at day 3, however we did not observe differences in Annexin-V expression on T cells following BLN stimulation (readout for AICD), possibly because AICD often peaks within 4-12 h of stimulation.

### BLN expands CD4^+^ and CD8^+^ but not γδ T cells from mixed PBMC cultures

**Figure 5:**
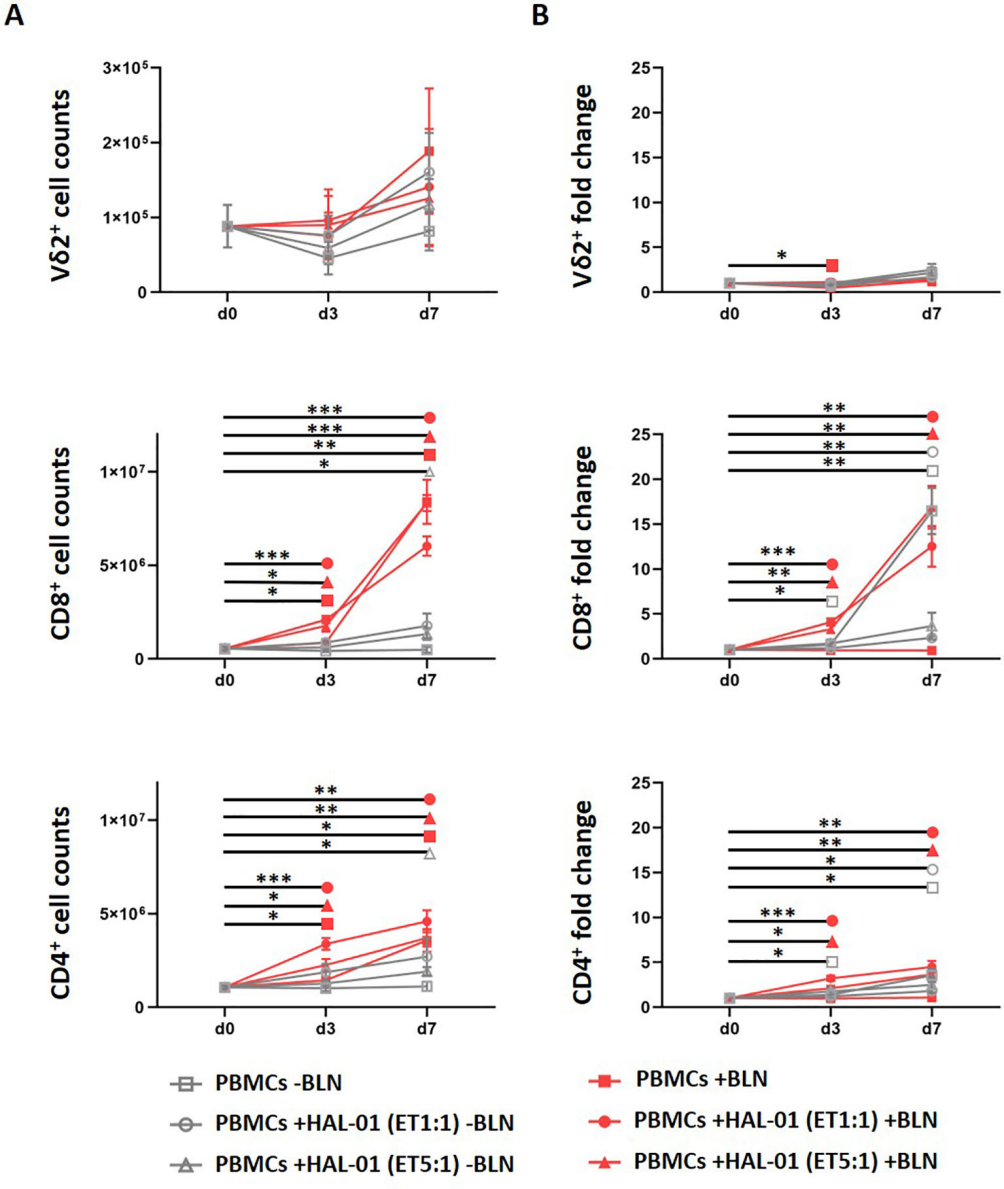
BLN-induced expansion of CD8 αβ but not γδ T cells in ex vivo cultures. **(A)** Absolute cell counts of Vδ2+ CD8+ and CD4+ T cells after three and seven days of PBMC stimulation with BLN (red) or without BLN (grey). Cultures without additional malignant B cells are marked as squares (containing autologous B cells, low CD19 load), cultures with medium tumor load (E:T 5:1) as triangles and high tumor load (E:T 1:1) as circles. **(B)** Fold change expansion of Vδ2+, CD8+ and CD4+ T cells after three and seven days. (n = 6). Data includes 6 matched healthy donors, depicted as mean value with SEM. Paired two−tailed t−tests were used for donor−matched comparisons and p−values were adjusted using Benjamini-Hochberg FDR across markers within each comparison. *p<0.05, **p<0.01, ***p<0.001.

Next, we asked whether BLN treatment in a mixed PBMC context could induce γδ T-cell proliferation alongside αβ T cells, despite unfavorable abundances of γδ T cells. This particular point is important in the clinical setting. To this end we stimulated PBMCs (n = 6) together with CD19^+^ HAL-01, at an E:T ratio of 1:1 (corresponding more to high CD19^+^ load when taking CD3 T-cell proportions of PBMCs into account), at an E:T ratio of 5:1 (medium CD19^+^ load) and without addition of CD19-expressing cell lines (low CD19^+^ load, autologous B-cells) for 7 days with (red) and without (grey) 20 ng/ml BLN (**Figure 5A**). We assessed absolute cell counts of CD4^+^, CD8^+^ and γδ T cells at day 0, day 3 and day 7 of the co-culture (**Supplementary Figure 7**) and assessed immune profiles of T cells. We noticed a significant expansion of CD4^+^ and CD8^+^ T cells in all three culture conditions with BLN treatment with low, medium and high CD19^+^ load. In contrast to CD4^+^ and CD8^+^ T cells, we did not see a noteworthy expansion of γδ T cells under BLN treatment from PBMCs up to day 7. As expected there was no significant increase in T-cell counts in the absence of BLN (**Figure 5**, grey lines). Interestingly we saw higher expansion rates of both CD4^+^ and CD8^+^ T cells in the presence of high target cell density compared to other two conditions with medium and low target cell density at day 3. At day 7, there were no differences among these conditions in CD4^+^ T cells. In CD8^+^ T cells however, the cultures with the highest CD19^+^ cells at baseline had the lowest CD8^+^ T-cell counts at day 7 compared to the other two cultures with medium and low frequencies of CD19^+^ cells (red filled mark). This was confirmed by strongest activation profile (CD25^+^, HLA-DR^+^) in the presence of high target cells (not shown), compared to other cultures, where EM CD8^+^ T cells predominated the BLN-containing cocultures on day 7, whereas the “naïve” population almost disappeared. In summary BLN application did not lead to γδ T-cell expansion in the presence of other T-cell populations, CD8^+^ T cells were the most expanded subset, as previously reported (53) and target load was inversely correlated with absolute CD8^+^ T-cell counts, suggestive of activation-associated contraction following strong activation.

### scRNA-seq reveals strong activation/exhaustion programs in αβ T cells

**Figure 6:**
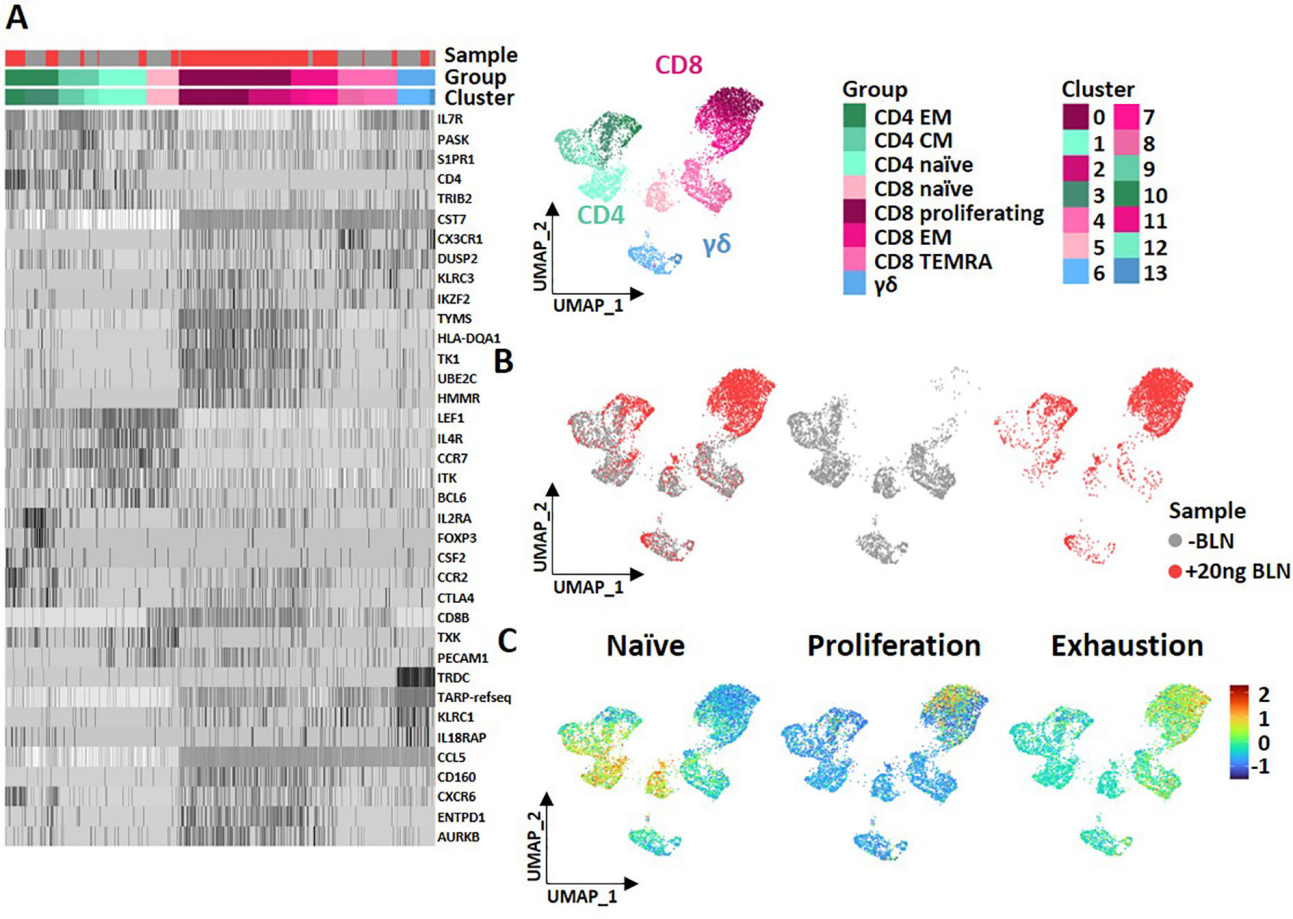
scRNA-seq reveals strong activation/exhaustion programs in αβ T cells. **(A)** Heatmap and UMAP 2D Space plot of 8 Clusters (groups) representing CD4+, CD8+ and γδ T cells and original 14 Clusters identified by Seurat clustering and corresponding samples with or without BLN are shown on top of the heat map. **(B)** UMAP 2D space plot of Seurat clustering representing CD3+ cells from the culture without (grey) and with 20 ng BLN (red). **(C)** UMAP 2D space plot highlights expression of genes representing naïve, proliferative and exhausted cells (respective gene sets are listed in **Supplementary Table 3**). Data obtained from one donor using a targeted T-cell gene panel.

To compare transcriptional profiles of αβ and γδ T cells following BLN stimulation at single-cell resolution, we performed targeted single-cell RNA sequencing of T cells from one healthy donor using the BD Rhapsody™ platform with a human T-cell panel focusing on key genes involved in T-cell responses. PBMCs from a healthy donor (containing autologous B cells) were cultured for 3 days with or without BLN (20 ng/ml). Subsequently, CD3^+^ T cells were negatively isolated for sequencing. In parallel, immunophenotyping was performed using AbSeq reagents and conventional flow cytometry to support cluster identification and validate phenotypic states (**Supplementary Figures 8A-D**). After quality control and removing non-viable cells, 6872 high-quality T cells were included in the analysis. Dimensionality reduction and Seurat-based clustering on UMAP space identified 14 initial clusters (**Supplementary Figure 8C**), which were manually curated and consolidated into eight main functional clusters (groups) based on top 5 characteristic genes (**Supplementary Table 4**, **Figure 6C**, **Supplementary Figure 8**). UMAP projection showed a clear separation between BLN-treated (red, *3, 576 cells*) and untreated (grey *3, 296 cells*) conditions, indicating major shifts in transcriptomic profiles upon BLN stimulation. UMAP projection shows minimal transcriptomic shifts in γδ T cells (predominantly Seurat clusters 6 and 13; **Figures 6A, B**, blue) following BLN exposure, with substantial overlap between -BLN and +BLN cells. In contrast, αβ T cells, especially CD8^+^ T cells, redistributed into BLN-enriched regions and upregulated proliferation/effector and exhaustion-associated signatures (**Figures 6B, C**). BLN stimulation led to increased expression of genes associated with proliferation and effector function, as well as exhaustion markers including TIGIT, TIM-3 (HAVCR2), LAG3, and CD160 (**Supplementary Figure 8B**). In contrast, γδ T-cell frequencies remained largely stable, consistent with the phenotypic data and aligned with *in vitro*, with lack of γδ T cell expansion from PBMCs at day 3 as shown in **Figure 5**. Of note again, also on transcript level, exhaustion marker TIGIT was not expressed on γδ T cells following BLN exposure (**Supplementary Figure 8B**) (54).

## Discussion

BLN is a highly potent bispecific T-cell engager directing T-cell-mediated cytotoxicity toward CD19-expressing malignant blasts and normal B cells. In this study, we dissected BLN-mediated cytotoxic mechanisms by directly comparing resting and activated CD3-expressing conventional αβ T cells (CD4^+^ and CD8^+^) and non-conventional γδ T cells, to evaluate their cytotoxic functions and phenotypic shifts following BLN stimulation.

Here we show that BLN efficiently redirected cytotoxicity toward CD19^+^ targets across αβ and γδ T-cell subsets. However, freshly isolated γδ T cells demonstrated reduced BLN-mediated cytotoxicity against CD19^+^ tumor cells compared to αβ T cells. Because BLN binds CD3ϵ rather than the TCR variable domains, differences between αβ and γδ T cells under BLN likely reflect CD3 epitope/clone biology and CD3 glycosylation. In addition, lineage-specific signaling might also play a role, as antigen stimulation of the γδ TCR itself does not trigger CD3 conformational change (CD3 CC), in contrast to αβ T cells (29, 55–57). In human Vγ9Vδ2 γδ T cells, effects of anti-CD3 clones differ markedly: UCHT1 clone enforces CD3 CC and strongly augments γδ cytotoxicity, whereas OKT3 clone is comparatively weak unless valency is increased or CD3 is deglycosylated (55). In BLN CD3 scFv derives from murine L2K 07 clone. Some older reviews have mentioned TR66 as the parental clone of anti−CD3 for BLN (58), but more recent primary sources identify L2K−07 as the basis (59–61). However, direct functional comparison of clone L2K 07 with UCHT1 and OKT3 in γδ T cells are still missing.

Among αβ T cells, CD8^+^ T cells mount a stronger response to BLN than CD4^+^ T cells, consistent with prior reports showing superior cytotoxic potency of CD8^+^ T cells in the presence of BLN (53). Collectively, these findings support notion that, *in vivo*, CD8^+^ T cells are the principal contributors to therapeutic responses to BLN. Given the low γδ T-cell frequencies in blood, these cells are readily outcompeted by αβ cells in mixed cultures, which likely contributed to the minimal γδ expansion observed ex vivo.

In contrast, γδ T-cell lines, activated and expanded ex vivo using Zole, which selectively expands the Vγ9Vδ2 γδ T-cell subset, exhibited comparable cytotoxic efficacy to PHA-expanded αβ T cells. Across experiments, antigen density and tumor burden emerged as dominant modulators of potency, and BLN dosing further tuned γδ T-cell activity. Both Zole-expanded γδ and PHA-expanded αβ T cells cleared CD19^+^ targets under low tumor burden (modelling MRD setting in ALL), whereas under high tumor burden (diagnosis/relapse−like) αβ T cells rapidly eliminated CD19^+^ targets, and γδ T-cell killing improved with higher BLN concentration over time (29). Other T−cell engager designs most likely constructed with the same CD3−binder lineage as BLN (62) (e.g., HER2×CD3) have shown potent γδ-mediated cytotoxicity *in vitro*. These experiments used much higher bispecific doses and higher effector−to−target ratios compared to conditions here (57, 62). These data do not suggest an intrinsic inability of γδ T cells to respond to CD3-engagers; rather, under the conditions tested, BLN elicited stronger responses in αβ than γδ T cells. They show potency of BLN-stimulated γδ T cells and that αβ cells respond more robustly to BLN than γδ cells, at the cost of a higher risk of overt activation. We showed that BLN triggered sustained surface CD3 down-modulation in αβ T cells but not in γδ T cells, particularly at high target-load and was accompanied by consistently higher sFas-L release in αβ cultures. High disease burden amplifies BLN-driven synapse frequency and CD3 signaling (40), which might predispose αβ T cells to early AICD. This level of TCR-CD3 engagement is known to induce CD3 down modulation (internalization and ζ chain degradation) and to trigger Fas/FasL mediated AICD on re-stimulation (45). Our observation of higher sFas-L secretion together with early CD3 down-modulation in αβ therefore is consistent with increased activation and suggests a greater susceptibility for Fas/FasL-associated apoptosis in αβ (particularly CD8^+^) T cells under high target load. However, AICD was not directly quantified in these experiments, and direct apoptosis measurements at early time points (e.g., Annexin V/active caspase-3) will be required to formally test this mechanism in future studies. Further, the preferential CD8 T-cell expansion at lower target load from co-cultures of activated T cells and mixed PBMC cultures supports this hypothesis. Clinically, the same principle underlies superior BLN outcomes at lower leukemia burden, the use of debulking, and BLN is frequently used in MRD settings and as a bridge to allogeneic transplantation (8, 12, 63, 64). This altogether suggests that high CD19 load accelerates αβ activation but limits CD8^+^ expansion, whereas Zole-expanded Vγ9Vδ2 γδ T cells retain CD3 and maintain surface CD3 under these conditions; whether this reflects reduced susceptibility to AICD requires direct testing.

Importantly day 14 Zole-expanded γδ T cells displayed an effector phenotype with low expression of inhibitory checkpoints. Conversely, PHA-expanded αβ T cells underwent rapid activation and differentiation from naïve to EM. Single-cell RNA−seq from one donor supported these findings: αβ cells acquired proliferative and activation/exhaustion programs, whereas γδ cells showed comparatively modest transcriptional shifts. Larger sample sets are required to validate these findings.

In our assays, γδ cultures while exerting cytotoxic effector functions, produced lower IFNγ and cytotoxic−granule release than αβ cultures and did not expand under BLN stimulation. Cytokine release syndrome (CRS) and Immune Effector Cell-Associated Neurotoxicity Syndrome (ICANS) are associated with systemic cytokine surges and widespread T-cell activation during CD3-engager therapy; However, our experiments were not designed to model these clinical toxicities, and cytokine levels and BLN exposure conditions *in vitro* may not reflect *in-vivo* exposure dynamics. Thus, these features may suggest a distinct inflammatory profile of γδ-based strategies. Whether γδ adoptive transfer alongside BLN therapy can deliver additional tumor control and how it impacts the incidence or severity of CRS/ICANS remains to be tested in future preclinical studies and clinical trials.

Zole expansion protocols have become a standard approach for generating clinical-grade γδ T-cell products (39). Several ongoing clinical trials (19) are evaluating Zole-expanded γδ T cells, both as monotherapy and in combination with BLN, to enhance treatment efficacy and improve T-cell fitness, especially in the context of heavily pretreated patients undergoing chemotherapy. A key advantage of γδ T cells in adoptive transfer settings is their innate-like, non-alloreactive profile, eliminating concerns related to GvHD, thus making them particularly attractive for allogeneic and off-the-shelf therapy. Taken together with the target-burden dependence observed here, combining adoptive γδ T-cell transfer with BLN may be most plausibly explored in low−burden/MRD-like settings and/or in patients with impaired αβ T-cell fitness; this hypothesis requires dedicated preclinical testing and clinical evaluation, as supported by preclinical CD19-BiTE and Vγ9Vδ2 γδ T-cell synergy (34). Additionally, optimizing γδ T-cell expansion protocols, for instance, incorporating cytokines IL-2, IL-15, and vitamin C, has been shown to significantly boost cytotoxic function and proliferation while reducing apoptosis, suggesting a promising direction for future clinical applications. This approach with 414 expanded γδ T-cell infusions into 132 cancer patients has been shown to be clinically safe without significant adverse effects and prolonged the survival of late-stage cancer patients who received ≥5 cell infusions in a small cohort of 8 liver and 10 lung cancer patients (32).

In conclusion, our findings emphasize the therapeutic potential of γδ T cells in adoptive T-cell therapies, particularly when expanded *ex vivo* with Zole (19, 39, 44). Their stable cytotoxic functionality, maintenance of CD3 expression under BLN stimulation, together with lack of alloreactivity, may offer advantages for the design of adoptive immunotherapy protocols, pending validation in patient-derived models and clinical studies. Future clinical studies are warranted to evaluate the clinical efficacy and long-term benefits of Zole-expanded γδ T-cell products alone or in combination with BLN and other immune modulators, to harness their full therapeutic potential and improve outcomes for patients with CD19-expressing malignancies.

## Data Availability

The original contributions presented in the study are included in the article/**Supplementary Material**. Further inquiries can be directed to the corresponding author.
