# Supplementary material for: Blinatumomab-driven T-cell activation in αβ and γδ T-cell subsets: insights from in vitro assays
Source: Front Immunol. 2026 Feb 6;17:1739493. doi: 10.3389/fimmu.2026.1739493 (PMC12920462; doi:10.3389/fimmu.2026.1739493)
Supplement: Supplementary file 1 [file DataSheet1.pdf]

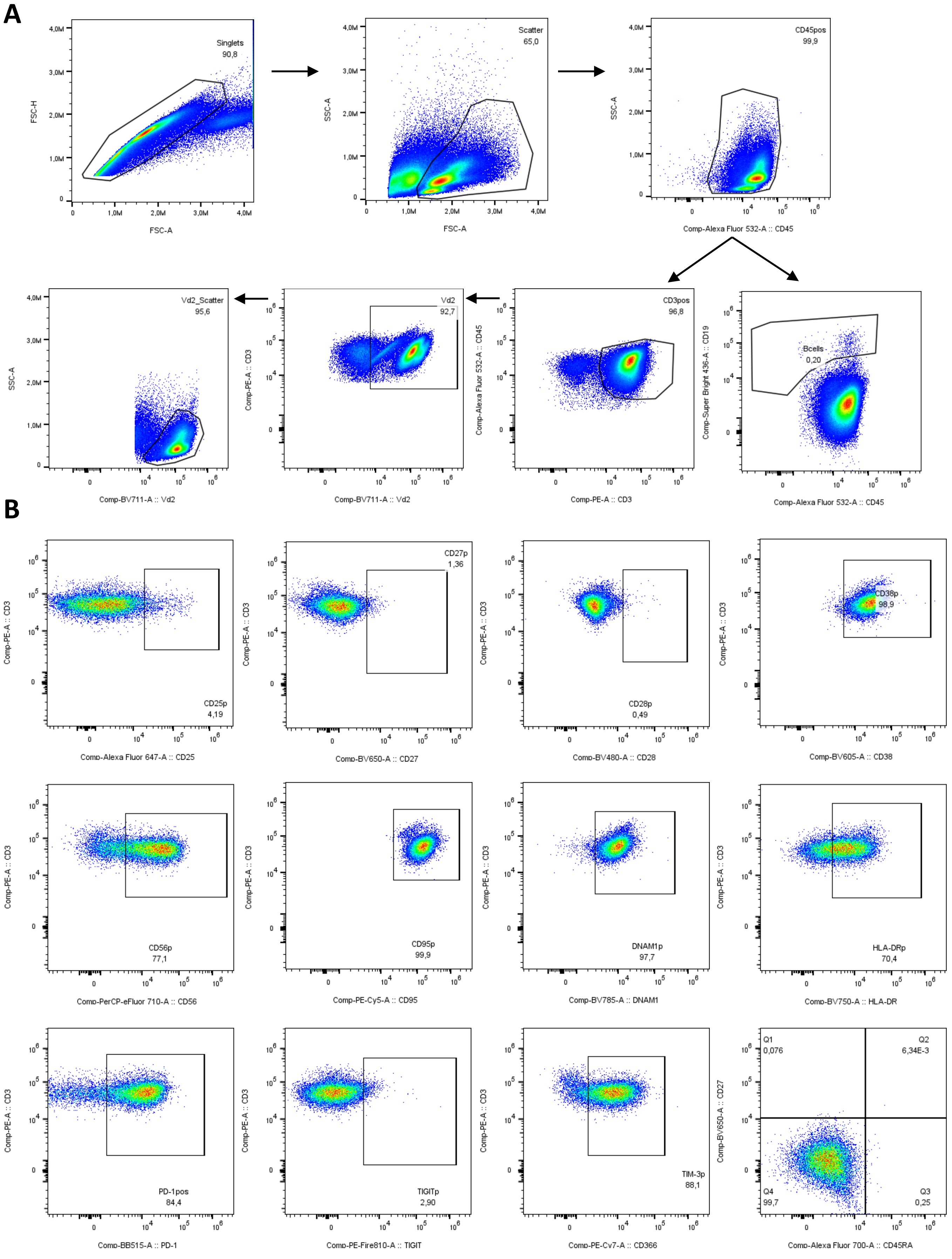

Supplemental Figure 1. Flow cytometry gating strategy for Zole-expanded γδ T-cell cultures. (A) Representative backbone gating (singlets, viable cells by FSC/SSC, lymphocytes by CD45-expression and T and B-cells. (B) Representative phenotyping gate for Vδ2 γδ T cells (TCRγδ+Vδ2+) and downstream subset/marker gates used for analyses.

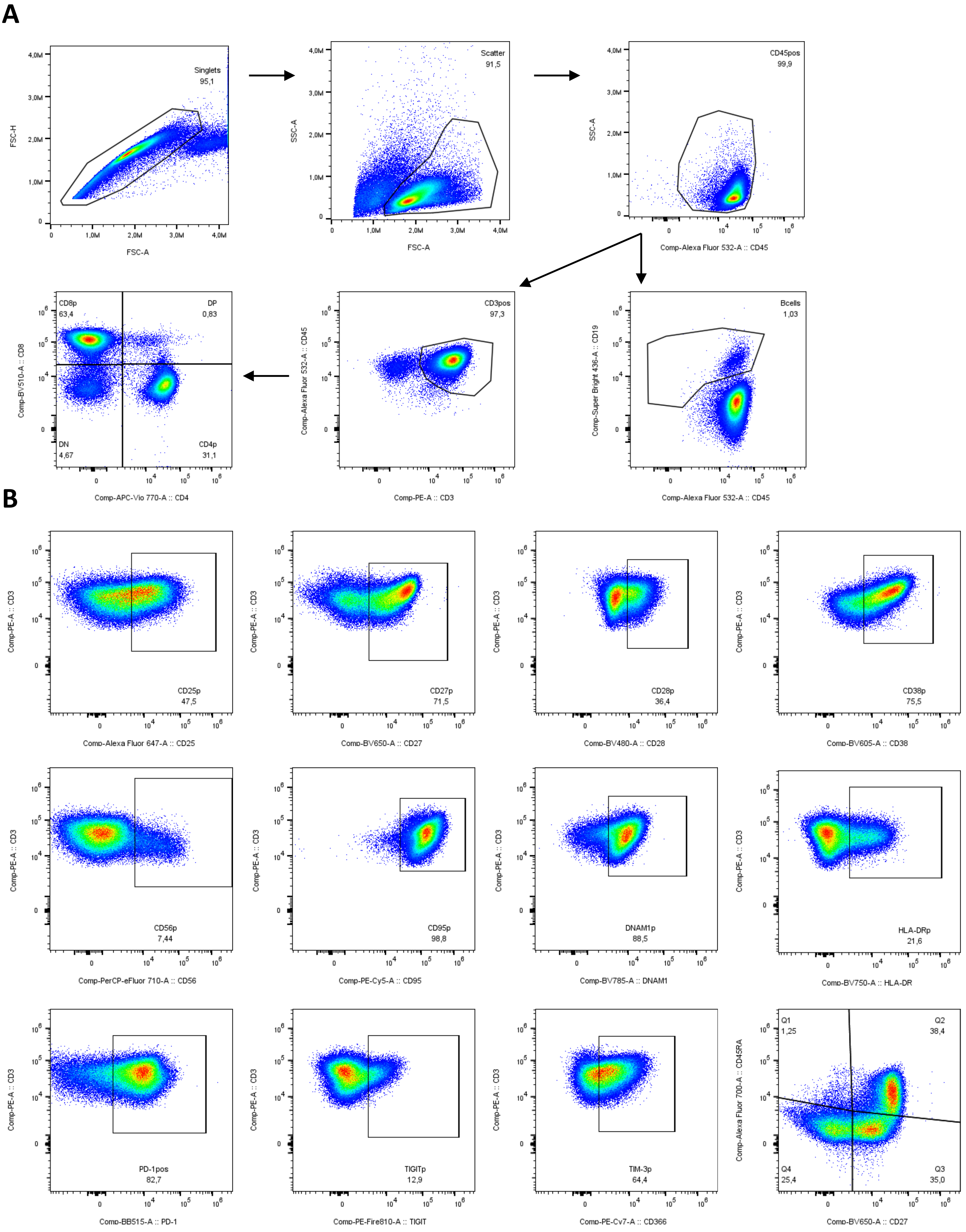

Supplemental Figure 2. Flow cytometry gating strategy for PHA-expanded αβ T-cell cultures. (A) Representative backbone gating (singlets, viable cells by FSC/SSC, lymphocytes by CD45-expression and T and B-cells. (B) Representative phenotyping strategy for CD8+ αβ T cells and downstream subset/marker gates used for analyses.

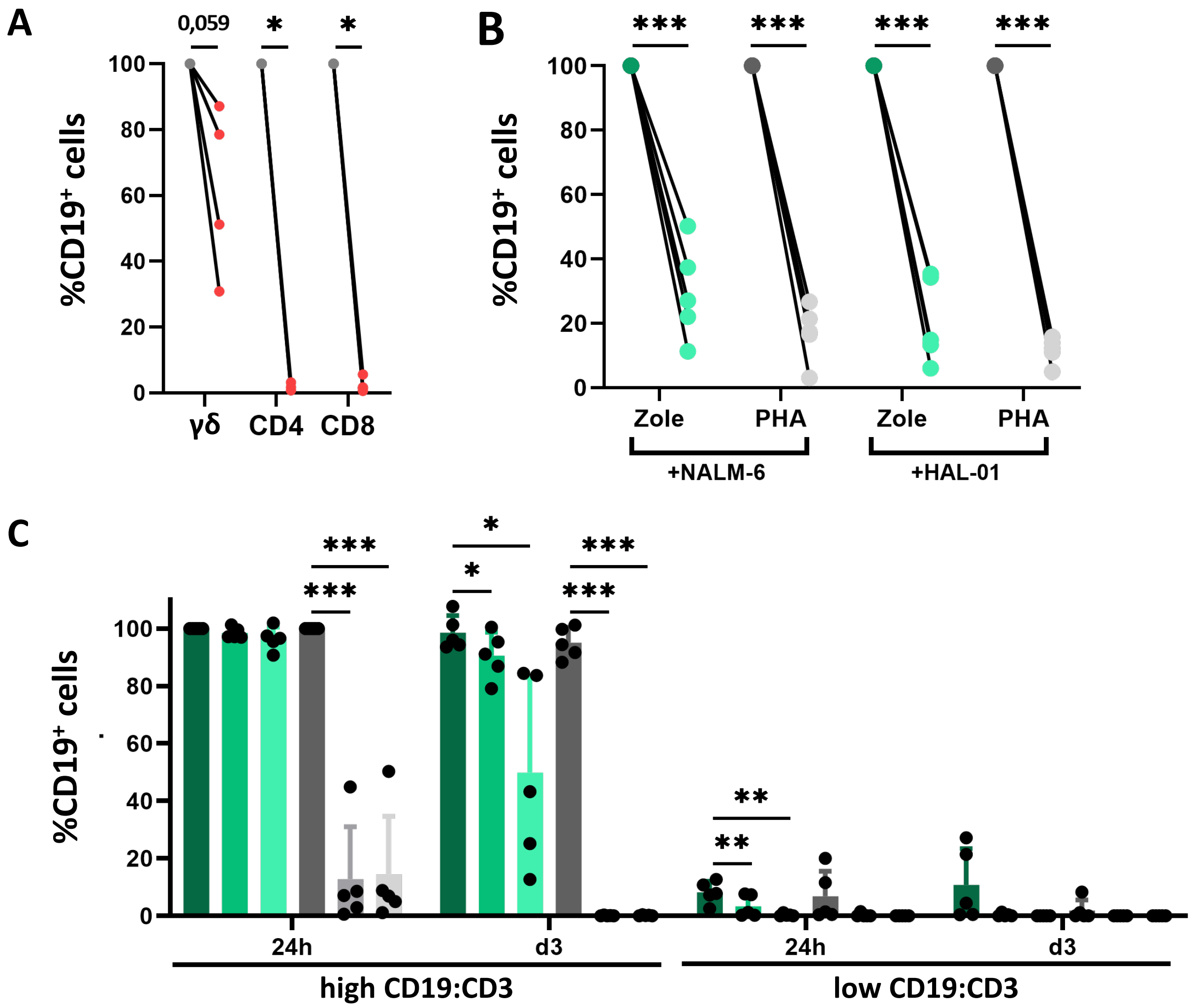

Supplemental Figure 3: BLN-specific lysis of CD19+ cells in in vitro cultures. Data correspond to Figure 1A (A), Figure 2A (B), and Figure 3A (C). For each donor, residual CD19+ cells in the −BLN condition were set to 100%, and the corresponding +BLN condition was expressed relative to this baseline: normalized residual CD19+ (%) = 100 × (residual CD19+% +BLN) / (residual CD19+% −BLN). (Equivalently, "BLN-specific lysis" can be derived as 100 − normalized residual CD19+ [%].) (A) Freshly isolated CD4+, CD8+, and γδ T cells co-cultured with HAL-01 for 3 days (n=4 donors). (B) PHA-expanded αβ and Zole-expanded γδ T-cell lines co-cultured with HAL-01 or NALM-6 for 24 h at low target burden (E:T 5:1; low CD19:CD3) (n=5 donors). (C) Expanded αβ and γδ T-cell lines co-cultured with HAL-01 under low target burden (E:T 5:1) and high target burden (E:T 1:5) and treated with BLN (0.5 or 20 ng/mL) for 24 h and 3 days (n=5 donors). Donor-matched ±BLN comparisons were analyzed using paired two-tailed t-tests. *p<0.05, **p<0.01, ***p<0.001.

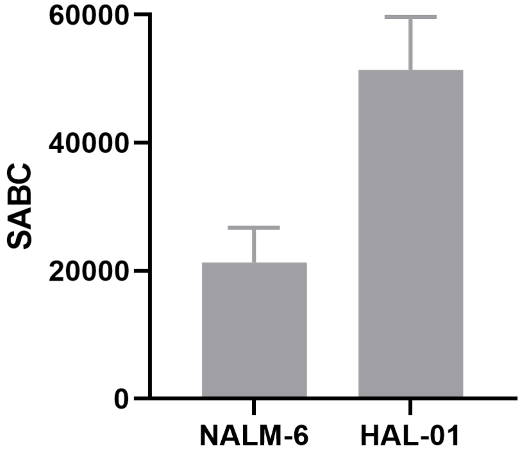

Supplemental Figure 4: Quantitative determination of CD19-antigen expression on B-cell surface of HAL-01 and NALM-6 cell lines as analyzed by flow cytometry using the purified anti-human CD19 antibody (clone HIB19) and QIFIKIT®. Shown are the median specific antibody binding capacity (SABC) values + 95% CI of four experiments.

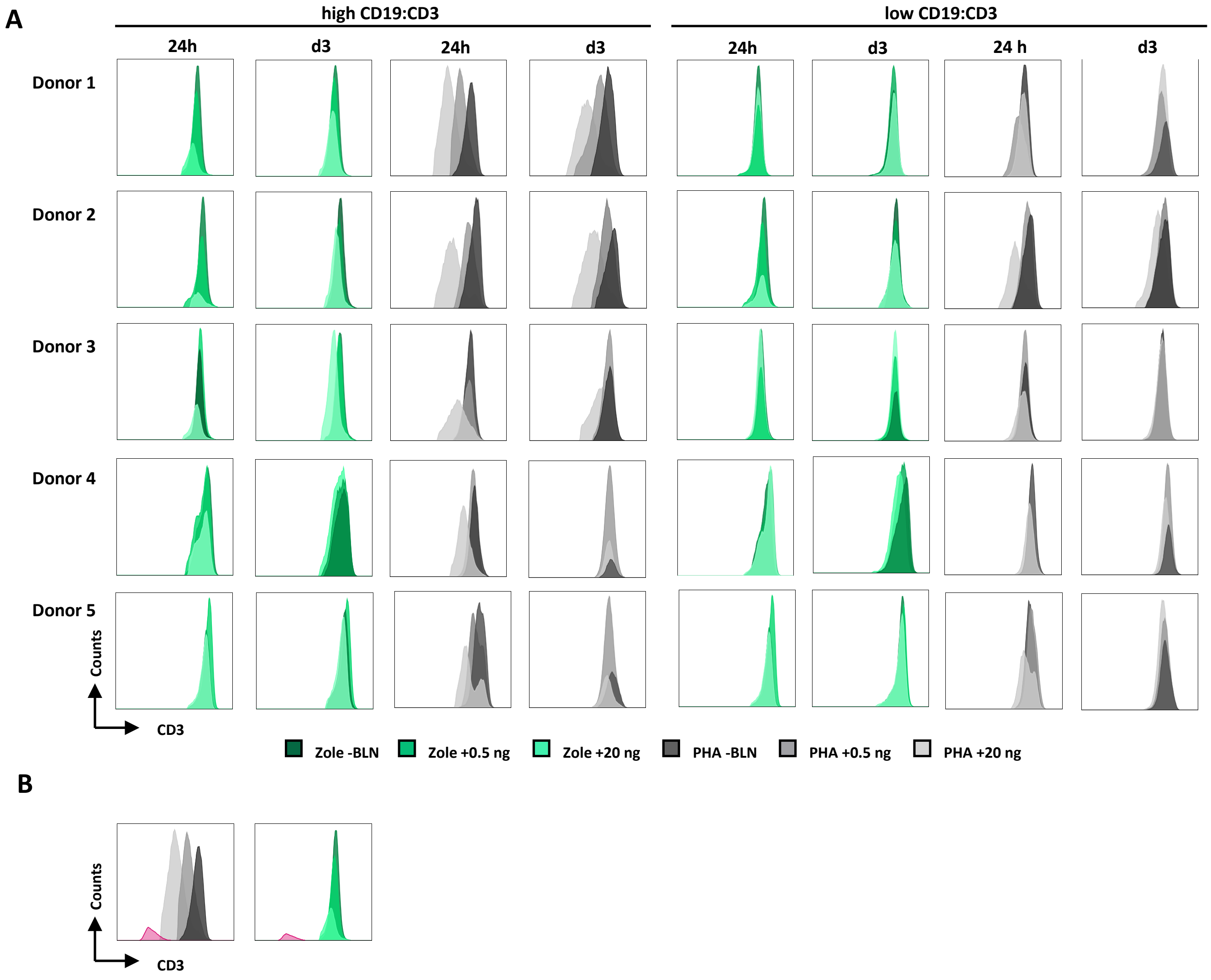

Supplemental Figure 5. CD3 down-modulation depends on CD19 target burden and is prominent in expanded αβ but not γδ T cells. (A) CD3 expression (MFI) in PHA-expanded αβ and Zole-expanded γδ T-cell cultures treated with BLN (0.5 or 20 ng/mL) under low target burden (E:T 5:1; low CD19:CD3) or high target burden (E:T 1:5; high CD19:CD3) after 24 h and 3 days (n=5 donors). (B) Representative CD3 histograms from PHA- or Zole-expanded cultures shown alongside the matched unstained control (pink).

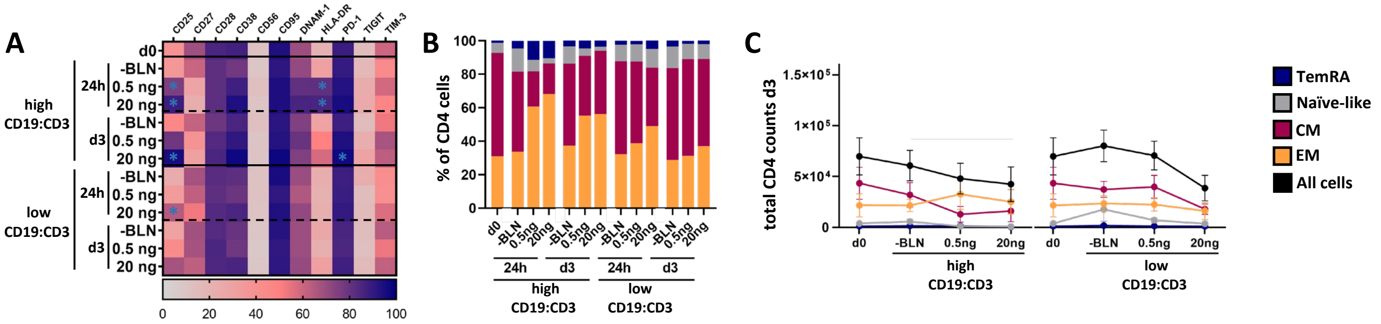

Supplemental Figure 6. Phenotypic changes in PHA-expanded CD4+ αβ T cells under varying BLN concentrations and CD19 target burden. (A) Marker expression of PHA-expanded CD4+ T cells at baseline (day 0) and after 24 h and 3 days of co-culture under low target burden (E:T 5:1) or high target burden (E:T 1:5), with BLN (0.5 or 20 ng/mL) or without BLN (n=5 donors). (B) Frequencies of CD4+ memory subsets (naïve, CM, EM, TEMRA), defined by CD27 and CD45RA. (C) Absolute counts of the corresponding subsets at day 0 and day 3 (mean ± SEM). Paired two-tailed t-tests were used for donor-matched comparisons between baseline and co-culture conditions and where multiple markers were tested, p-values were adjusted using Benjamini–Hochberg FDR. *p<0.05.

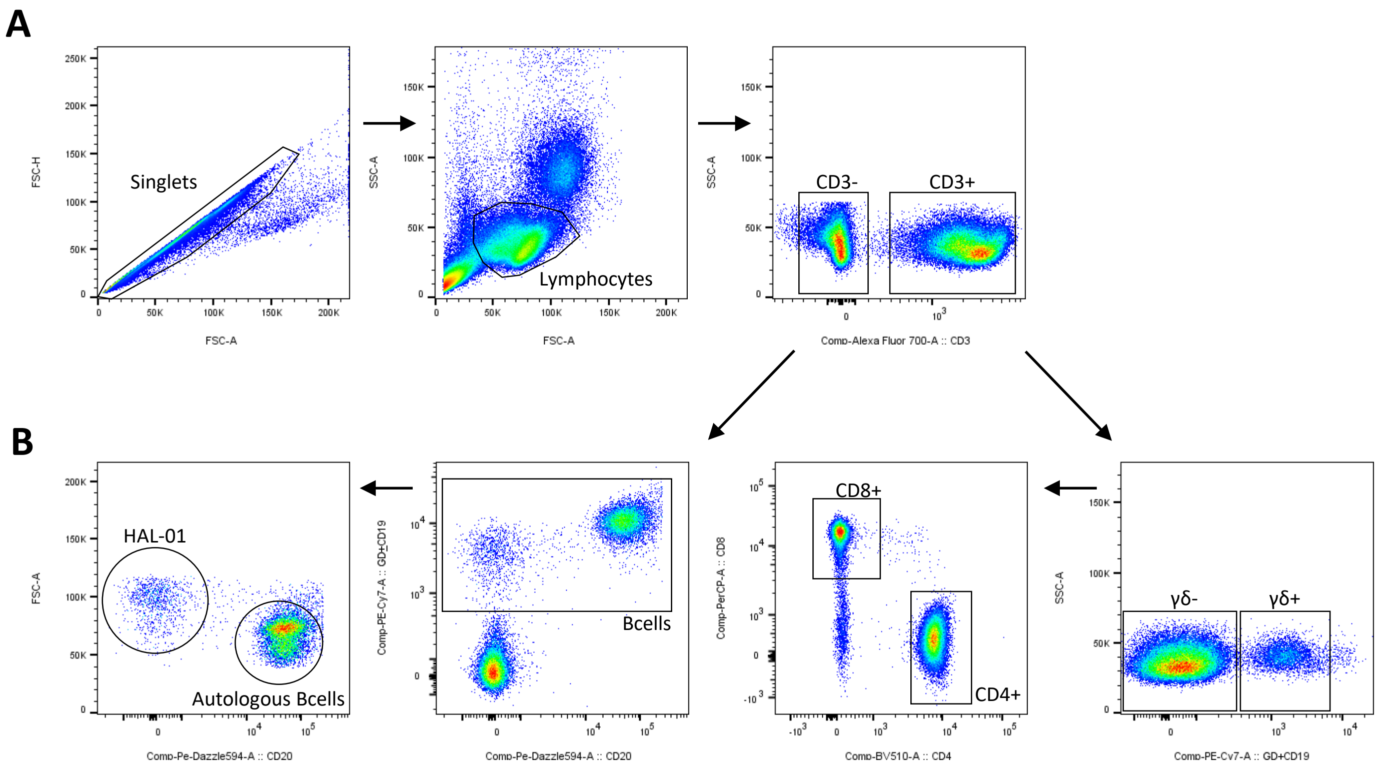

Supplemental Figure 7. Flow cytometry gating strategy for PBMCs co-cultures. (A) Representative backbone gating used throughout the study (Singlets, lymphocytes by FSC/SSC, live cells), followed by identification of CD3+ T-cell subsets and target populations (HAL-01 cells and CD19+ B cells).

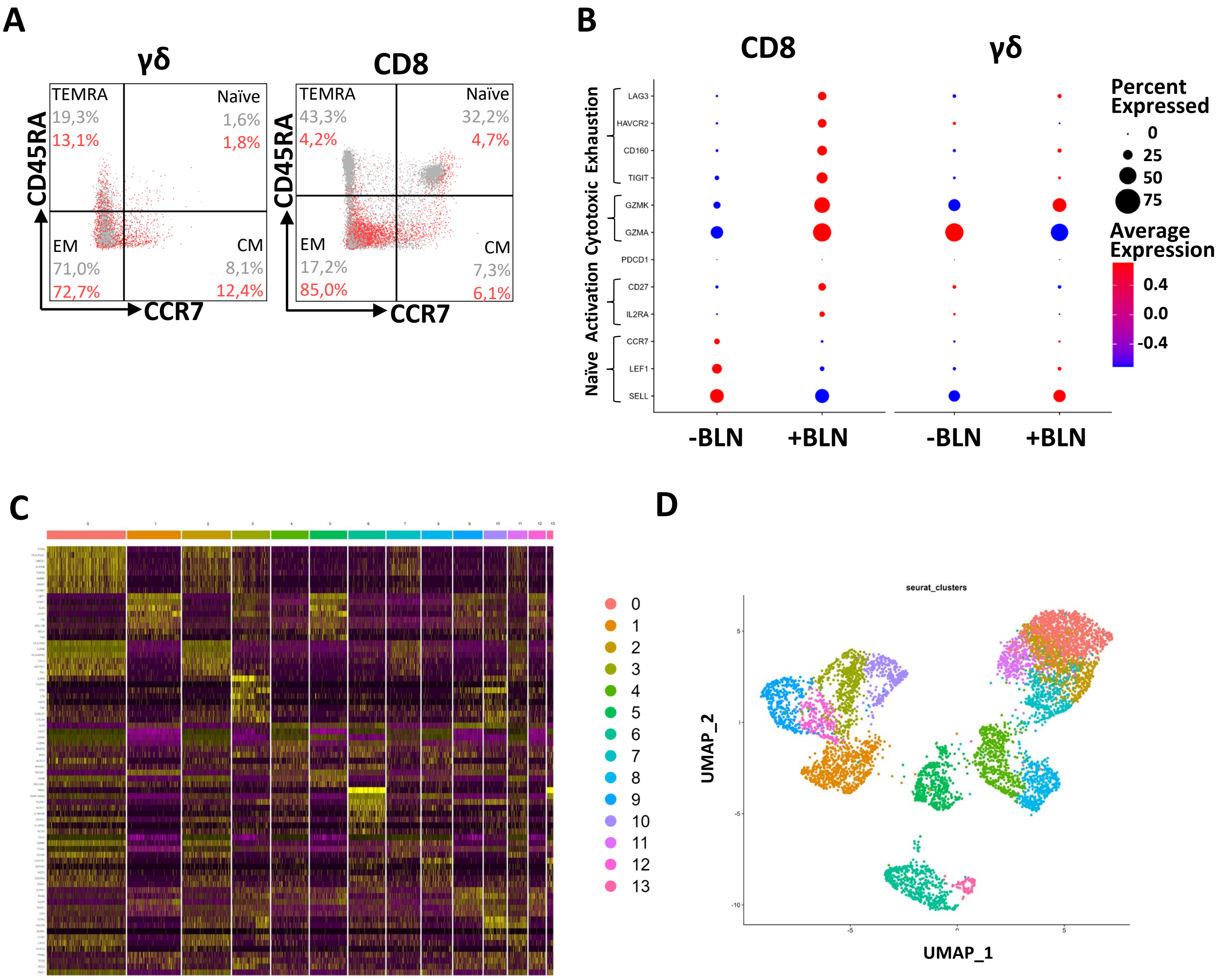

Supplemental Figure 8. Supportive analyses for targeted single-cell RNA-seq of BLN-stimulated PBMC cultures (single donor). PBMCs from one healthy donor were stimulated in vitro ± BLN prior to CD3+ T-cell isolation and targeted scRNA-seq. (A) Flow-cytometric immunophenotyping of γδ and CD8+ T cells in treated vs untreated cultures; naïve, CM, EM, and TEMRA subsets were distinguished by CCR7 and CD45RA. (B) Dot plot of selected T-cell fitness/exhaustion-associated genes in CD8+ and γδ T cells; dot size indicates the percentage of cells expressing the gene and dot color indicates average expression. (C–D) UMAP visualization of Seurat-derived clustering (14 initial clusters) prior to manual curation, annotated as: 0: CD8$^+$. proliferating; 1: CD4$^+$ naïve; 2: CD8$^+$ proliferating; 3: CD4$^+$ EM; 4: CD8$^+$ TEMRA; 5: CD8$^+$ naive; 6: γδ$^+$; 7: CD8$^+$ EM; 8: CD8$^+$ TEMRA; 9: CM; 10: CD4$^+$ EM; 11: CD8$^+$ EM; 12: CD4$^+$ CM; 13: γδ$^+$
